# *Erigeron annuus* Extract Improves DNCB-Induced Atopic Dermatitis in a Mouse Model via the Nrf2/HO-1 Pathway

**DOI:** 10.3390/nu16030451

**Published:** 2024-02-03

**Authors:** Myeongguk Jeong, Hyeokjin Kwon, Yeeun Kim, Hyunwoo Jin, Go-Eun Choi, Kyung-Yae Hyun

**Affiliations:** 1 Department of Biomedical Laboratory Science, College of Health Sciences, Catholic University of Pusan, Busan 46252, Republic of Korea; audrnr04@gmail.com (M.J.); ghy8627@gmail.com (H.K.); yeeun0509@naver.com (Y.K.); jjinhw@cup.ac.kr (H.J.); 2 Next-Generation Industrial Field-Based Specialist Program for Molecular Diagnostics, Brain Busan 21 Plus Project, Graduate School, Catholic University of Pusan, Busan 46252, Republic of Korea; 3 Department of Clinical Laboratory Science, Dong-Eui University, Busan 47340, Republic of Korea

**Keywords:** *Erigeron annuus*, subacute toxicity, atopic dermatitis, antioxidant, Nrf2, HO-1

## Abstract

Atopic dermatitis (AD) is a persistent inflammatory skin condition resulting from an intricate interplay among genetic, immunological, and environmental factors. *Erigeron annuus* (EA), an annual winter plant belonging to the family Asteraceae, possesses anti-inflammatory, cytoprotective, and antioxidant activities. In this study, we hypothesized that *Erigeron annuus* extract (EAE) could be an effective agent for ameliorating AD-like symptoms. To confirm this hypothesis in vitro, we used H_2_O_2_-stimulated human keratinocytes (HaCaT cells) to demonstrate that pre-treatment with EAE protected against oxidative stress. HaCaT cells pretreated with EAE and stimulated with H_2_O_2_ showed decreased intracellular malondialdehyde content, increased superoxide dismutase activity, and reduced intracellular reactive oxygen species accumulation. To verify the in vivo hypothesis based on the intracellular results, an AD disease mouse model was induced with 1-chloro-2,4-dinitrobenzene (DNCB), and EAE was orally administered at a non-toxic concentration according to the toxicity evaluation results. The results showed that AD disease models in BALB/c mice exhibited reduced ear epidermal thickness, scratching behavior, and mast cell infiltration. In conclusion, our results indicate that EAE has the potential to improve AD by upregulating the nuclear factor erythroid 2-related factor 2 (Nrf2)/heme oxygenase-1 (HO-1) signaling pathway.

## 1. Introduction

Atopic dermatitis (AD) is one of the most prevalent chronic inflammatory skin diseases worldwide, affecting all races and age groups [1]. AD is caused by a complicated interplay between genetic, immunological, and environmental factors, resulting in a wide range of clinical manifestations [2]. AD pathogenesis includes the impairment of skin barrier function, immune dysregulation, allergic sensitization, and increased oxidative stress due to the presence of reactive oxygen species (ROS) [3,4,5,6]. These factors contribute to severe itching, dryness, and bacterial skin infections [7,8]. AD, which presents as pruritic and erythematous skin lesions, is frequently characterized by relapse and can negatively affect the quality of life of patients [9,10,11,12,13,14]. At present, the worldwide prevalence of AD ranges from approximately 1% to 20% and is steadily increasing in developed countries [15,16]. Topical steroids are widely used to treat AD. However, long-term use of topical steroids results in specific adverse reactions, including flushing, skin atrophy, and suppression of the hypothalamic–pituitary–adrenal (HPA) axis [17,18,19]. Therefore, the development of safe and effective AD treatment supplements for all age groups is necessary.

Folk remedies are being used worldwide to treat and alleviate health problems [20]. The World Health Organization approximates that around 80% of the population in developing countries relies on traditional remedies, especially those derived from plant extracts, as their primary healthcare solution [21]. The extensive utilization of traditional medicines has raised concerns about scientific documentation regarding the safety, effectiveness, side effects, and toxicological profiles associated with these treatments [20,22]. Therefore, the increasing reliance on traditional folk medicine and the exploration of new natural product candidates for the treatment of serious diseases have made the study of the chemical toxicology of medicinal plants a major concern [23,24,25]. Traditional medicines are extensively used to treat serious conditions, such as fever, pain, and diabetes, and in certain instances, conditions such as cancer, hepatitis, various skin ailments, and liver diseases. This underscores the importance of elucidating the chemotoxicological profiles of these traditional remedies [26,27].

Several natural products ameliorate inflammatory skin conditions and prevent oxidative stress [28,29,30]. *Erigeron annuus* (EA) is a naturalized annual winter plant that belongs to the Asteraceae family [31]. EA has strong vitality and reproductive ability and grows in degraded land, fallow land, and roadsides. It has been studied for its anti-inflammatory, protective, and antioxidant activities [32,33,34]. Despite various studies, there is no subacute toxicity evaluation following the guidelines of the Organization for Economic Cooperation and Development (OECD) for assessing the toxicological characteristics of EA that have been reported. These guidelines, recommended by international regulatory organizations, are critical for conducting research and clinical trials on humans.

Oxidative stress occurs due to an excess of free radicals, ROS, and changes in the antioxidant defenses [35,36]. Under pathological conditions, the overproduction of ROS leads to cellular damage through processes such as lipid peroxidation, protein oxidation, and DNA modification [37]. Acting as the primary defense against damage caused by ROS, superoxide dismutase (SOD) catalyzes the conversion of peroxides into oxygen and hydrogen peroxide. One of the markers of tissue damage caused by oxidative stress in vivo is malondialdehyde (MDA), the end product of lipid peroxidation [38]. When exposed to severe oxidative damage, cells activate an antioxidant protection network organized by key regulatory factors including nuclear factor erythroid 2-related factor (Nrf2) and heme oxygenase-1 (HO-1) [39,40]. Activation of the Nrf2/HO-1 pathway alleviates AD-like symptoms [41,42,43]. The activation of the Nrf2/HO-1 pathway induces the expression of antioxidant enzymes and aims to restore redox homeostasis [44].

While numerous studies have demonstrated the anti-inflammatory and cytoprotective effects of EA [34,45], no study has directly investigated the therapeutic effects of EA in AD disease models. In this study, we hypothesized that EA could be an effective agent for alleviating AD. The aim of this study was to confirm these hypotheses and identify the related mechanisms. We first verified the protective effect of EA against oxidative damage caused by H_2_O_2_ in HaCaT cells, confirmed the therapeutic effect of EA in an AD disease model induced by DNCB, and investigated the underlying mechanism. We also evaluated the toxic concentrations of EA, for which no data are currently available.

## 2. Materials and Methods

### 2.1. Preparation of Erigeron annuus Extracts (EAE)

The extraction process was carried out with a modification of a method that has been described previously [46]. A dried whole EA plant was purchased from Daum International (Hanam, Republic of Korea). The whole plant (1 kg) was powdered and boiled in distilled water at a 1:10 ratio for 12 h at 80 °C. The extract was then filtered and concentrated using an R100 + B100 rotary vacuum evaporator (Lab Scitech, Corona, CA, USA) The concentrated extract was freeze-dried for 72 h with a freeze dryer (Ilshin Lab, Yangju, Republic of Korea) and stored in vacuum packages at 4 °C until use.

### 2.2. Cell Culture

HaCaT cells were grown in Dulbecco’s modified Eagle medium (DMEM) (GIBCO, Paisley, UK) containing 10% fetal bovine serum (GIBCO, Paisley, UK), 100 U/mL penicillin, and 100 µg/mL streptomycin. Cells were incubated in a culture environment with a temperature of 37 °C with 5% CO_2_.

### 2.3. Cell Viability

To determine the cytotoxicity of EAE, HaCaT cells were exposed to different concentrations of EAE and cell viability was assessed using Cell Counting Kit-8 (CCK-8; Dojindo, Kumamoto, Japan). Cells were plated at a concentration of 1 × 10^5^ cells/well in a 96-well plate and cultured for 24 h at 37 °C with 5% CO_2_. After 24 h of incubation, various concentrations of extract (0, 50, 100, 200, 400, 600, and 800 μg/mL) were added and incubated for 24 h. Then, a 10 μL CCK-8 solution was added and allowed to react in an incubator for 30 min. The absorbance was then assessed at a wavelength of 450 nm using a Varioskan LUX microplate reader (Thermo Fisher Scientific, Waltham, MA, USA).

### 2.4. In Vitro Antioxidant Activity

The 2,2-diphenyl-1-picrylhydrazyl (DPPH) radical-scavenging assay was used to evaluate the in vitro antioxidant activity of the EAE. Ascorbic acid at the same concentration was used as a control. The DPPH solution was dissolved in ethanol and used at a concentration of 200 μm, and various concentrations of EAE (2 mL) and DPPH solution (2 mL) were reacted for 30 min in the dark. Following the reaction, absorbance was assessed at 517 nm using a Varioskan LUX microplate reader. Please see Equation (1):

(1) $$\mathrm{DPPH}\;\mathrm{scavenging}\;\mathrm{ratio}\;(\%)=\left(1-\frac{A_{i}-A_{t}}{A_{c}}\;\right)\times 100\%$$

where $A_{i}$ is the absorbance of the sample, $A_{t}$ is the absorbance of the sample solvent or ethanol, and $A_{c}$ is the absorbance of DPPH and the sample solvent.

### 2.5. Establishment of the H_2_O_2_-Induced Oxidative Stress Model

HaCaT cells were plated at a concentration of 1 × 10^6^ cells/well in a 6-well plate and cultured for 24 h at 37 °C with 5% CO_2_. To establish the H_2_O_2_-induced oxidative stress model, the culture medium was replaced, and HaCaT cells were exposed to different concentrations of H_2_O_2_ for a duration of 3 h. H_2_O_2_ was diluted in DMEM without fetal bovine serum. The concentrations of H_2_O_2_ used were 0, 0.1, 0.2, 0.4, 0.6, 0.8, 1, 1.2, 1.4, and 1.6 mM. As a control, HaCaT cells without H_2_O_2_ were used. Cell viability was assessed with CCK-8. Following the addition of 10 μL of CCK-8 solution to each well, the cells were incubated for 30 min at 37 °C in a 5% CO_2_ environment. The absorbance was measured at 450 nm using a Varioskan LUX microplate reader. For subsequent experiments, the H_2_O_2_ concentration that resulted in 50% cell viability was selected.

### 2.6. Protective Effect of EAE on HaCaT Cells by H_2_O_2_-Induced Oxidative Stress

We investigated the protective properties of EAE in mitigating oxidative stress induced by H_2_O_2_. Untreated normal cells were used as controls. HaCaT cells were seeded at a concentration of 1 × 10^6^ cells/well in a 6-well plate. HaCaT cells from the H_2_O_2_ group were treated with varying concentrations of EAE for 24 h. After 24 h, the medium was removed, and the cells were treated with H_2_O_2_ (1.2 mM) for 4 h. Cell viability was measured using the CCK-8.

### 2.7. Analysis of MDA and SOD Contents in HaCaT Cells

HaCaT cells were plated at a concentration of 1 × 10^6^ cells/well in a 6-well plate, cultured for 24 h, treated with various concentrations of EAE, and cultured for an additional 24 h. Following the cultivation period, the medium was extracted, and the cells underwent treatment with H_2_O_2_ at a concentration of 1.2 mM for 4 h. The supernatant was removed, and the cells underwent three washes with Dulbecco’s phosphate-buffered saline (DPBS). The cells were lysed with lysis buffer, collected, and centrifuged to collect the whole-cell lysate. Intracellular MDA and SOD activities were then measured using assay kits, according to the manufacturer’s instructions (Biomax, Guri, Republic of Korea).

### 2.8. Measurement of ROS Production

Intracellular ROS production was assessed using the 2′,7′-dichlorofluorescin diacetate (DCFH-DA) fluorescent probe. Cells were exposed to DPBS with a concentration of 10 μM of DCFH-DA and then incubated at 37 °C for a duration of 30 min. After removing the fluorescent probe (DCFH-DA), the cells underwent two washes with DPBS and were visualized using a fluorescence microscope (DP71, Olympus, Tokyo, Japan). The fluorescence intensity of each cell was measured as the average cell fluorescence intensity using ImageJ software v.1.52 (National Institute of Health, Bethesda, MD, USA).

### 2.9. Animals

Male BALB/c mice, six weeks old, were purchased from KOSA BIO (Sungnam, Republic of Korea). All animals were raised under specific pathogen-free (SPF) conditions and maintained in an individually ventilated cage (IVC) laboratory with a temperature of 23 ± 2, a 12 h light/dark cycle, and 50 ± 5% humidity. Standard diet and water were provided without restriction. Approval for all experimental procedures was granted by the Institutional Animal Care and Use Committee of the Catholic University of Pusan (CUP AEC 2022-004).

### 2.10. Sub-Acute Toxicity (28-Day Repeat Dose Toxicity)

Sub-acute toxicity testing was conducted in accordance with guideline no. 407 of the OECD with slight modifications [47]. In this experiment, 30 BALB/c mice (15 males, 15 females) were used and randomly divided into three groups according to sex (5 males and 5 females per group). After one week of acclimatization, the control group was orally administered distilled water. The low-dose group was orally administered 500 mg/kg EAE. The high-dose group was orally administered 1000 mg/kg EAE. All groups were dosed daily for 28 days, and on day 29, the animals from all groups were euthanized and necropsied. Subsequently, all the tissues and organs were visually inspected and weighed.

### 2.11. Body Weight and Organ Weight Measurement and Serum Biochemistry Analysis

After 28 days of administration and 12 h of fasting, the mice were anesthetized with a combination of alfaxalone (25 mg/kg, Careside, Seongnam, Republic of Korea) and xylazine (5 mg/kg, Bayer Korea, Seoul, Republic of Korea). After anesthetizing the mice, blood was collected via cardiac puncture. For hematological analysis, blood samples were collected in ethylenediaminetetraacetic acid (EDTA) tubes. Hematological analyses were performed using a DxH 500 Hematology Analyzer (Beckman Coulter, Miami, FL, USA). For the analysis of blood biochemical parameters, plasma obtained after centrifugation of the collected blood at 2500× *g* for 15 min was used. Biochemical analysis was executed utilizing an automated biochemistry analyzer, the BT1500 (Biotecnica Instrument S.p.A., Rome, Italy).

Changes in the animals’ body weights were measured using an electronic balance (Ohaus, Parsippany, NJ, USA) immediately after oral administration of EAE once every three days. For organ weights, the liver, spleen, kidneys, heart, and lungs were removed after euthanizing the animals and weighed using an electronic balance.

### 2.12. Atopic Dermatitis Model Induction and Treatment

After the mice were acclimatized for one week, DNCB (1-chloro-2,4-dinitrobenzene, Sigma-Aldrich, St. Louis, MO, USA) was dissolved in an acetone–olive oil suspension (4:1) at a concentration of 1% and applied to the right ear three times a week. Thereafter, they were sensitized with a 0.4% DNCB solution three times a week while on EAE and dexamethasone. An acetone–olive oil (4:1) suspension without DNCB was applied to the normal group. The mice were grouped into the normal group (normal), DNCB group (DNCB), EAE 500 mg/kg group with DNCB, EAE 1000 mg/kg group with DNCB, and DNCB group with dexamethasone (DEX). Each group consisted of six mice. The EAE 500 mg/kg and 1000 mg/kg groups received EAE diluted in sterile distilled water to 500 and 1000 mg/kg, respectively, which was administered orally daily. The DEX group received dexamethasone diluted to 1 mg/kg in distilled water, which was administered orally daily. The control and DNCB groups received distilled water orally daily.

### 2.13. Measures Body Weight and Scratching Frequency

The body weights of all mice were recorded every three days using an electronic balance after treatment with an acetone–olive oil suspension with or without DNCB. The scratching frequency was measured after all treatments. First, the mice were allowed to acclimate within the cage for 1 h, and then the scratching frequency was measured and recorded for 10 min [48]. Three researchers were randomly assigned to groups and measured it three times. To distinguish it from grooming, we counted only the scratching behavior of the right ear.

### 2.14. Histological Analysis

After euthanizing the mice, ear tissue was collected, fixed with 10% neutral formalin, and subsequently processed to create paraffin blocks. The paraffin blocks were cut into sections with a thickness of 4 μm. These sections underwent staining with hematoxylin and eosin (H&E) for the purpose of measuring the thickness of the ear epithelium. For mast cell analysis, the sections were subjected to Toluidine Blue staining, visualized, and examined under a microscope. The tissue sections were observed and analyzed utilizing a DMi1 microscope (Leica, Wetzlar, Germany). The examination of morphology was conducted utilizing ImageJ v1.53 (National Institutes of Health, Bethesda, MD, USA) and presented as a bar graph. All histological analyses were performed on three different sections.

### 2.15. Western Blotting

Ear tissues were homogenized with protein Extraction Solution (iNtRON Bio, Seongnam, Republic of Korea) that included protease/phosphatase inhibitors (GenDEPOT, Katy, TX, USA) and were centrifuged at 12,000 rpm at 4 °C for 15 min to collect whole proteins from the supernatant. Protein concentrations were assessed and quantified through the use of a bicinchoninic acid (BCA) Protein Assay Kit (iNtRON Bio, Seongnam, Republic of Korea). Proteins were subjected to 10% sodium dodecyl sulfate–polyacrylamide gel electrophoresis and transferred onto polyvinylidene fluoride (PVDF) membranes. The PVDF membranes were blocked with 3% bovine serum albumin. The PVDF membrane, after being blocked, underwent an overnight incubation at 4 °C with the primary antibody. Subsequently, it was exposed to a goat anti-rabbit secondary antibody conjugated with horseradish peroxidase for 1 h. After washing the PVDF membrane, the protein bands were amplified with an enhanced chemiluminescence (ECL) kit, and the membrane was observed using a ChemiDoc XRS+ system (Bio-Rad, Hercules, CA, USA).

### 2.16. Statistical Analysis

Data from at least three independent experiments are presented as mean ± standard deviation (SD). Statistical analyses were performed using GraphPad Prism 6 software (GraphPad Software Inc., San Diego, CA, USA) version 6.01. Post hoc Bonferroni tests and one-way ANOVA analysis of variance were used for multiple comparisons. A statistical *p*-value below 0.05 was considered statistically significant.

## 3. Results

### 3.1. Antioxidant Activity of EAE against H_2_O_2_-Induced Oxidative Stress

**Figure 1:**
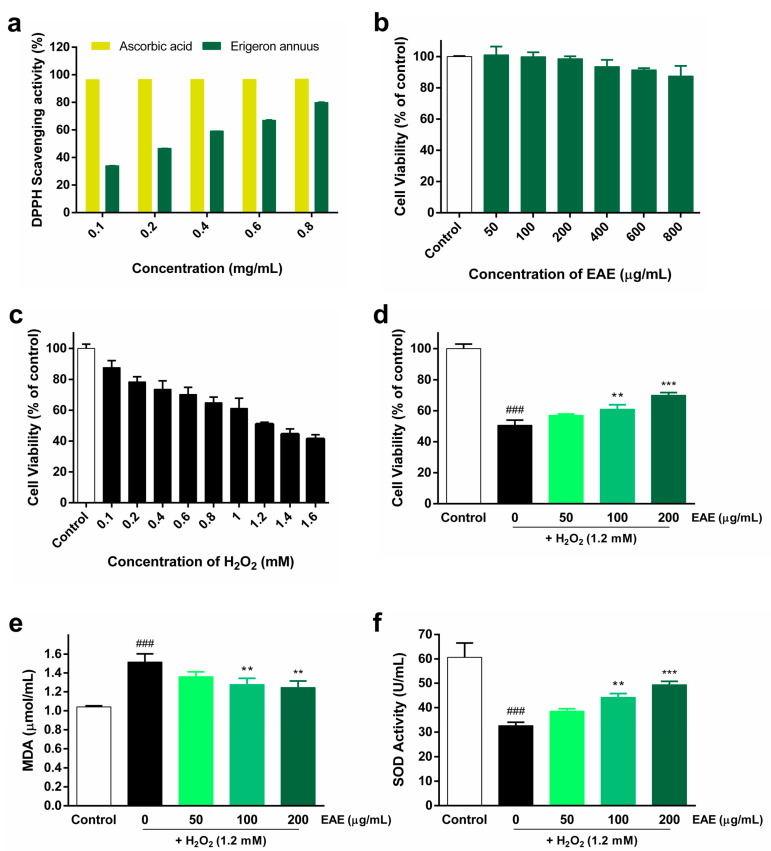
Antioxidant activity of *Erigeron annuus* extract (EAE) and its impact in the H_2_O_2_-induced HaCaT oxidative stress model. (**a**) 2,2-Diphenyl-1-picrylhydrazyl (DPPH) radical-scavenging assay of different concentrations of EAE. (**b**) Cytotoxicity and optimal concentrations were measured after 24 h of EAE treatment in HaCaT cells. (**c**) To establish a model of H_2_O_2_-induced oxidative stress, HaCaT cells were exposed to various concentrations of H_2_O_2_ for a duration of 4 h. (**d**) Cells were pretreated with various concentrations of EAE for 24 h and then exposed to H_2_O_2_ for 4 h. Cell viability was analyzed using the cell counting kit 8 (CCK-8) assay. Cells were pretreated with various concentrations of EAE for 24 h and treated with H_2_O_2_ for an additional 4 h. Intracellular (**e**) malondialdehyde (MDA) content and (**f**) superoxide dismutase (SOD) activity in whole-cell lysates were quantified using commercial assay kits following the guidelines provided by the manufacturer. Experiments were performed in triplicate, and the bar graphs represent the mean ± standard deviation (SD). Significant difference compared to the control group: ### *p* < 0.001, and compared to H_2_O_2_-only treatment: ** *p* < 0.01; *** *p* < 0.001.

The DPPH radical-scavenging activity of EAE, cytotoxicity of EAE in HaCaT cells, treatment of HaCaT cells with various concentrations of H_2_O_2_, and protective effect of EAE against H_2_O_2_-induced oxidative stress are shown in Figure 1. The DPPH radical-scavenging assay was used to measure the antioxidant activity of EAE. EAE showed a concentration-dependent increase in DPPH radical-scavenging activity. At 0.8 mg/mL EAE, the DPPH radical-scavenging rate was 79.95% (Figure 1a). To evaluate the toxicity of EAE, HaCaT cells were treated with different concentrations (50, 100, 200, 400, 600, and 800 μg/mL) of EAE for 24 h. Subsequently, we analyzed survival using the CCK-8 kit and found that it began to affect survival at 400 µg/mL (Figure 1b). In order to use EAE at a non-toxic concentration, we used a concentration of 200 µg/mL with cell viability above 95%. To establish a cellular model of H_2_O_2_-induced oxidative stress, HaCaT cells were treated with different concentrations (0.1, 0.2, 0.4, 0.6, 0.8, 1, 1.2, 1.4, and 1.6 mM) of H_2_O_2_ and incubated for 4 h. The survival rate of HaCaT cells during H_2_O_2_ treatment decreased with increasing H_2_O_2_ concentrations (Figure 1c). The viability of HaCaT cells treated with 1.2 mM H_2_O_2_ was 51.14% of that of the control. The viability of HaCaT cells treated with 1.2 mM of H_2_O_2_ treatment was reduced to 51.14% compared to that of the control, establishing a model of H_2_O_2_-induced oxidative stress in HaCaT cells, and 1.2 mM H_2_O_2_ was used in subsequent experiments. The protective effect of EAE on H_2_O_2_-induced HaCaT cell viability was analyzed using a CCK-8 kit. As the concentration of EAE increased, cell viability significantly increased in a concentration-dependent manner compared with treatment with H_2_O_2_ alone (Figure 1d). MDA expression levels were the highest in the H_2_O_2_ treatment group, and pretreatment with EAE significantly reduced MDA expression levels in a concentration-dependent manner compared to the H_2_O_2_-alone treatment group (Figure 1e). SOD activity was the lowest in the H_2_O_2_ treatment alone, and pretreatment with EAE significantly increased SOD activity in a concentration-dependent manner compared to the H_2_O_2_-alone treatment (Figure 1f). Thus, the results show that EAE significantly reduced H_2_O_2_-induced oxidative stress.

### 3.2. Protective Effect of EAE on H_2_O_2_-Induced ROS Production in HaCaT Cells

**Figure 2:**
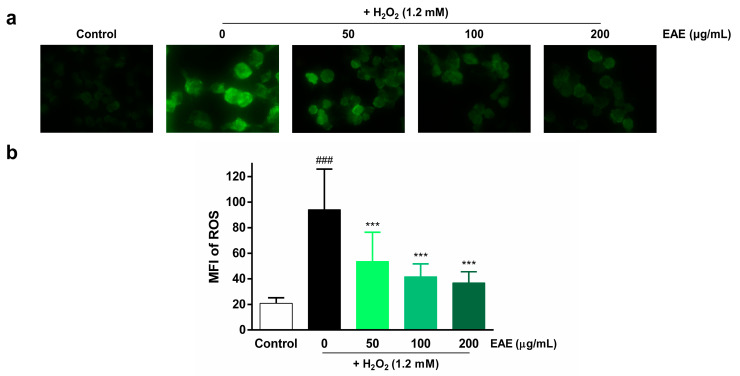
Effect of *Erigeron annuus* extract (EAE) on intracellular reactive oxygen species (ROS) production in H_2_O_2_-induced HaCaT cells. (**a**) The cells underwent pretreatment with different concentrations of EAE for a duration of 24 h followed by exposure to H_2_O_2_ for 4 h. Intracellular ROS were then visualized under a 400× fluorescence microscope using 2′,7′-dichlorofluorescin diacetate (DCFH-DA). (**b**) Intracellular ROS production was quantified using the program ImageJ. Experiments were performed in triplicate, and the bar graphs represent the mean ± standard deviation (SD). Significant difference compared to the control group: ### *p* < 0.001, and compared to H_2_O_2_-only treatment: *** *p* < 0.001.

To assess the protective effects of EAE on H_2_O_2_-induced ROS production in HaCaT cells, we examined intracellular ROS production by utilizing DCFH-DA. Intracellular ROS levels were visualized using fluorescence microscopy (Figure 2a) and quantified using ImageJ software v.1.52 (Figure 2b). The highest fluorescence intensity was observed in the H_2_O_2_ treatment group compared to the control, indicating that H_2_O_2_ induced intracellular ROS production. However, pre-treatment with EAE (50, 100, 200 µg/mL) significantly decreased intracellular ROS production in a concentration-dependent manner.

### 3.3. Subacute Oral Toxicity Studies

#### 3.3.1. Assessment of Body Weight and Major Organ Weights

**Figure 3:**
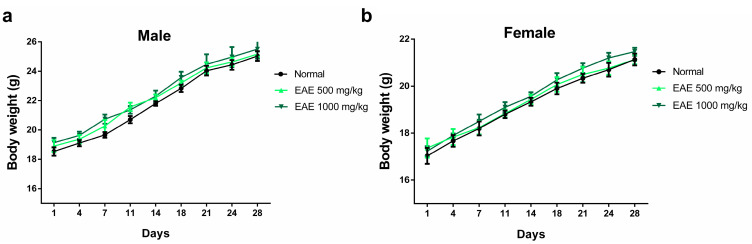
Effect of repeated 28-day oral dosing of *Erigeron annuus* extract (EAE) on body weight in (**a**) male and (**b**) female mice. The normal group treatment (distilled water) and EAE (500 and 1000 mg/kg) were administered orally. There was no significant (*p* > 0.05) difference in body weight between the three groups.

The oral subacute toxicity study of EAE was performed according to OECD guideline 407 with minor modifications [47]. No mortality or other unusual behavioral changes were observed in any group at the end of the 28-day oral dosing period. Body weight changes were measured immediately after the oral administration of EAE every 3 days. After 28 days of treatment, there was no significant difference in body weight among the three groups (Figure 3). After 28 days of oral administration, the mice were necropsied on day 29 and the major organs of male and female mice were weighed. No significant changes in the weights of major organs (heart, liver, spleen, kidney, and lung) were observed in males and females compared with those in the normal group (Table 1).

#### 3.3.2. Hematological Parameters

The hematological parameters of each group are summarized in Table 2. Hematopoietic parameters are highly susceptible to harmful chemicals and serve as important indicators of physiological and pathological conditions in both humans and animals [49]. The main hematological parameters analyzed were white blood cells (WBCs), lymphocytes (LY), monocytes (MO), neutrophils (NE), eosinophils (EO), red blood cells (RBCs), platelets (PLTs), hemoglobin (HGB), hematocrit (HCT), mean corpuscular volume (MCV), mean corpuscular hemoglobin (MCH), and mean corpuscular hemoglobin concentration (MCHC). The PLT of males and females in the EAE treatment group showed a slight decrease; however, the difference was not statistically significant. No changes in hematological parameters were observed after EAE administration in males or females compared to the normal group.

#### 3.3.3. Biochemical Parameters

Table 3 provides a summary of the biochemical parameters for each group. The key biochemical parameters examined included alkaline phosphatase (ALP), alanine aminotransferase (ALT), aspartate aminotransferase (AST), total bilirubin (TB), total protein (TP), albumin (ALB), triglycerides (TG), total cholesterol (TC), and lactate dehydrogenase (LDH). No statistically significant alterations were observed in the biochemical parameters of both male and female subjects in the EAE-treated group when compared to those in the normal group. These results suggest that treatment with EAE at concentrations of 500 and 1000 mg/kg did not cause toxicity. The non-toxic concentrations of 500 and 1000 mg/kg EAE were used in further studies.

### 3.4. Effect of Oral Administration of EAE on Mice

**Figure 4:**
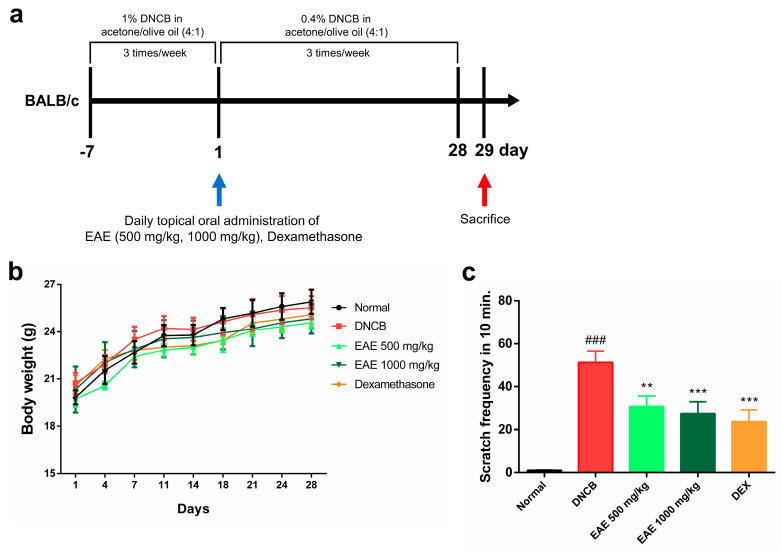
Effect of oral administration of *Erigeron annuus* extract (EAE) on symptoms of atopic dermatitis (AD) disease model induced by DNCB. (**a**) Schematic of the AD induction and experimental protocol. (**b**) Weight changes in all groups were measured three times a week using electronic scales. (**c**) After acclimatizing the mouse to the cage, the frequency of scratching only the right ear was measured and recorded for 10 min. Experiments were performed in triplicate, and the bar graphs represent the mean ± standard deviation (SD). Significant difference compared to the normal group: ### *p* < 0.001, and compared to DNCB group: ** *p* < 0.01; *** *p* < 0.001.

We investigated whether oral administration of EAE could affect symptomatic relief in an AD model. During the experimental period, the normal group received an acetone–olive oil suspension (4:1) without DNCB, and the experimental group received an acetone–olive oil suspension (4:1) with DNCB, three times a week. The treatment group received EAE or DEX orally daily for 28 days (Figure 4a). No significant changes in body weight were observed in any of the groups during the study period (Figure 4b). The scratching behavior was assessed for 10 min after all treatments to determine whether itching was reduced by DNCB application. The scratching frequency exhibited a significant decrease in the EAE group compared to the DNCB group, and this reduction was concentration-dependent (Figure 4c). These results indicate that administration of EAE alleviated DNCB-induced AD.

### 3.5. Histologic Analysis of AD Disease Model Induced by DNCB and the Impact of EAE on the Nrf2/HO-1 Signaling Pathway

**Figure 5:**
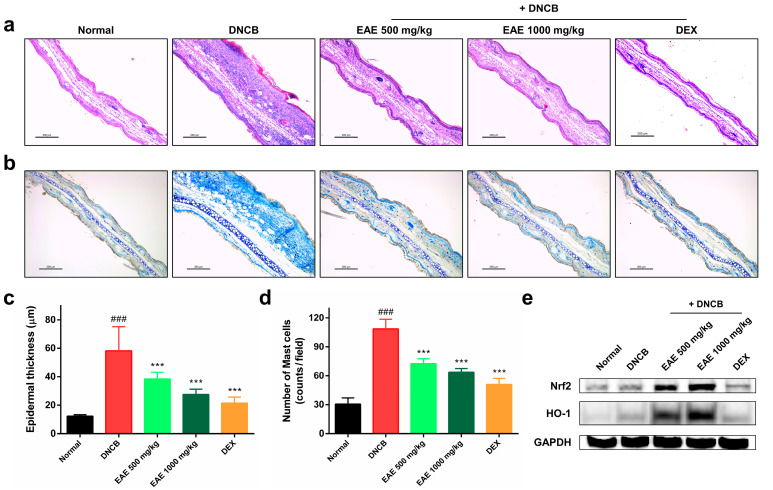
Effects of oral administration of *Erigeron annuus* extract (EAE) on epidermal thickness, mast cell infiltration, and activation of Nrf2/HO-1 signaling pathway in the atopic dermatitis (AD) disease model induced by DNCB. (**a**) Sections of mouse ear tissue were subjected to hematoxylin and eosin (H&E) staining for the purpose of assessing epidermal thickness. Scale bar: 200 μm. (**b**) Toluidine blue staining was utilized to observe the infiltration of mast cells in the mouse ear tissue. Scale bar: 200 μm. (**c**) Epidermal thickness of mouse ear tissue was measured using ImageJ at three randomly selected sites on H&E-stained slides. (**d**) Mast cell infiltration in mouse ear tissue was determined by counting mast cells in three randomly selected areas on slides stained with toluidine blue. (**e**) Nrf2 and HO-1 protein expression in mouse ear tissue was evaluated by Western blot. Experiments were performed in triplicate, and the bar graphs represent the mean ± standard deviation (SD). Significant difference compared to the normal group: ### *p* < 0.001, and compared to DNCB group: *** *p* < 0.001.

After all the experiments were completed, the mice were euthanized, and an ear tissue slide was prepared. H&E staining was performed to measure ear epithelium thickness. Epidermal thickness was determined using ImageJ software. Repeated application of DNCB to the mouse ear skin resulted in hyperkeratosis and an increase in the epidermal thickness of the ear, which was significantly reduced by EAE treatment in a concentration-dependent manner (Figure 5a,c). We conducted toluidine blue staining to visualize the infiltration of mast cells. The number of infiltrating mast cells in the dermal layer increased after repeated DNCB application. Treatment with the EA extract significantly reduced mast cell infiltration in a concentration-dependent manner (Figure 5b,d). Western blotting was performed to investigate the effects of EAE on the Nrf2/HO-1 signaling pathway in DNCB-induced AD mice. Dexamethasone, a corticosteroid, did not significantly change the expression of Nrf2 and HO-1. In conversely, EAE treatment resulted in a significant dose-dependent increase in Nrf2 and HO-1 expression (Figure 5e). These results demonstrate that the antioxidant and anti-inflammatory effects of EAE are exerted by activating the Nrf2/HO-1 pathway.

## 4. Discussion

Atopic dermatitis (AD) is a common chronic inflammatory skin disease. Corticosteroids such as dexamethasone for the treatment of AD have a number of side effects associated with their long-term use. Because of these side effects, new treatments for AD are constantly being investigated. Therefore, it is necessary to develop plant-derived drugs with minimal side effects for the treatment of AD. AD is a common chronic inflammatory skin disease. Corticosteroids used to treat AD, such as dexamethasone, are well known to have long-term side effects. Numerous studies have attempted to identify alternative treatments using plant-derived drugs. However, alternative treatments without side effects are needed for chronic inflammatory skin diseases.

In this study, we focused on investigating the antioxidant properties of EA. EA has been shown to be an antioxidant and neuroprotective agent, and various studies have demonstrated its anti-atherosclerotic, neuroprotective, antioxidant, and cytoprotective activities [32,33,45]. Therefore, in this study, we hypothesized that the antioxidative properties of EA could serve as an alternative therapeutic approach for AD. The aim of this study was to test this hypothesis. We first confirmed the protective effect of EA against oxidative damage caused by H_2_O_2_ in HaCaT cells, confirmed the therapeutic effect of EA in a DNCB-induced AD model, and investigated the underlying mechanism. We also evaluated the toxic concentrations of EA for which no data are currently available.

DPPH is effective for evaluating the antioxidant effects of substance extracts, and evaluation of the antioxidant activity of plant compounds or extracts is important for investigating functional factors [50]. This study is the first to report that EAE exhibits a dose-dependent increase in antioxidant capacity in vitro, using a radical-scavenging assay.

Keratinocytes form the epidermis of the skin and contribute to AD [51]. HaCaT cells serve as a model for studying oxidative stress responses associated with skin problems in vitro. We investigated the effects of EAE on the regulation of oxidative stress in HaCaT cells induced by H_2_O_2_ in vitro. In this study, HaCaT cells injured by H_2_O_2_ treatment showed a concentration-dependent decrease in cell viability, with approximately 50% survival at 1.2 mM H_2_O_2_, thereby establishing an oxidative stress model. The concentration-dependent reduction in cell viability by H_2_O_2_ treatment is consistent with previous findings [52,53,54]. Furthermore, stimulation of HaCaT cells with H_2_O_2_ resulted in an excessive increase in MDA production and a significant decrease in SOD activity. However, pretreating the cells with EAE significantly reduced the generation of MDA caused by H_2_O_2_ stimulation in a concentration-dependent manner and significantly increased SOD activity in a concentration-dependent manner. DCFH-DA was used to investigate the ROS-scavenging activity of EAE, and intracellular ROS production in HaCaT cells was measured. DCFH-DA, which is absorbed through the cell membrane and is transformed to DCFH by cellular esterases, and intracellular ROS are converted to fluorescently active DCF, which can be visualized using fluorescence microscopy [55]. Intracellular ROS content was significantly increased by H_2_O_2_ treatment, whereas pretreatment with EAE significantly decreased intracellular ROS content in a concentration-dependent manner.

Prior to the animal model experiments, we conducted tests to evaluate the toxicity of EA. Reports on the toxicity evaluation of EA have not been published to date, and toxicity studies should be conducted to determine its safety. Furthermore, the WHO emphasizes validation through scientific studies for the safe use of natural products [56]. Therefore, to select a safe, non-toxic dose, the subacute oral toxicity of EAE was evaluated in animal models according to OECD guideline 407 [47]. For subacute oral toxicity evaluation, 500 and 1000 mg/kg EAE were administered daily for 28 days. Clinical events, such as signs of toxicity and death, were monitored immediately after daily EAE administration. No signs of distress, abnormal behavioral changes, or death were observed in any of the groups during the 28-day dosing period. No abnormal changes in weight were observed. This indicates that 28 days of EAE administration did not affect mouse growth. No significant differences were observed between the major organ weights of the treatment and normal groups. Hematological parameters (WBC, LY, MO, NE, EO, RBC, PLT, HGB, HCT, MCV, MCH, and MCHC) 28 days after EAE administration were not significantly different in the EAE-treated group compared to those in the vehicle group, indicating no toxic effects on circulating blood cells or blood cell production. It did not cause anemia in any of the groups, supporting its non-toxicity. Similarly, the biochemical parameters of the mice were not significantly different in the EAE group when compared to the normal group, demonstrating that the EAE did not alter the common markers of renal and hepatic toxicity (ALP, ALT, AST, LDH, TB, TG, and TC) and did not affect the kidneys or liver. As a conclusion of the subacute toxicity evaluation, oral administration of EAE at concentrations of 500 and 1000 mg/kg for 28 days did not result in significant toxicity or death. Therefore, based on the subacute toxicity evaluation, the EAE concentrations of 500 and 1000 mg/kg were used for further anti-AD studies.

AD and oxidative stress are closely related [57]. In a study by Devadasanet et al. comparing blood oxidant and antioxidant levels in pediatric patients with AD, it was found that the levels of lipid oxidation products were significantly higher in AD cases compared to healthy controls [38]. Furthermore, the accumulation of ROS beyond the antioxidant defense system causes oxidative stress and contributes to the development of AD and skin diseases [58]. AD is a persistent inflammatory skin disease in which immune cells, including macrophages and mast cells, are recruited to the lesions [59,60]. Symptoms of AD commonly include increased epidermal thickness, edema, and erythema [61]. In a previous study, we reported that an AD disease model induced by DNCB in BALB/c mice resembled human AD, including hemorrhages, epidermal hypertrophy, and IgE-activated mast cell infiltration [62,63]. In our study, the repeated application of DNCB in an AD disease model induced erythema, hyperkeratosis, increased epidermal thickness, mast cell infiltration, and itching [64]. Based on the antioxidant activity of the EAE, we investigated its anti-AD effects in vivo. Despite repeated application of DNCB to the ear, oral administration of EAE significantly decreased ear epidermal thickness compared to DNCB alone, decreased scratching behavior, and alleviated DNCB-induced AD effects, such as reduced mast cell infiltration.

Nrf2 is a transcription factor sensitive to redox changes and has been implicated in various inflammatory conditions [65]. This transcription factor is activated through the antioxidant-responsive element (ARE) pathway in response to oxidative stress, resulting in the transcriptional activation of numerous antioxidant genes, including HO-1 [66]. The Nrf2/HO-1 pathway is triggered by oxidative stress and inflammation to maintain cellular homeostasis [67,68]. Furthermore, the Nrf2/HO-1 signaling pathway acts as a protective mechanism regulating chronic inflammatory responses triggered by oxidative stress [69,70,71]. Earlier research has reported that activation of the Nrf-2/HO-1 pathway improves symptoms of AD induced by DNCB [72,73]. Our results suggest that the oral administration of EAE exerts anti-inflammatory and protective impacts on skin cells by reducing the production of pro-inflammatory cytokines and chemokines by keratinocytes via the Nrf2/HO-1 signaling pathway. Consequently, EAE has the potential to improve skin lesions in AD models by restoring skin homeostasis through the activation of the Nrf2/HO-1 pathway, alleviation of oxidative stress, and inhibition of inflammation in a mouse model. Taken together, this study demonstrated the anti-inflammatory effect of EAE and its underlying mechanisms through in vitro and in vivo experiments on AD. These results indicate its potential as an alternative treatment for AD. However, additional research is necessary to identify unidentified active molecules and elucidate their interrelated mechanisms of action.

## 5. Conclusions

In the present study, the antioxidant and anti-AD effects of EAE were evaluated in vitro and in a mouse model. EAE significantly reduces H_2_O_2_-induced oxidative stress in HaCaT cells in a concentration-dependent manner. DNCB induced an AD disease model in BALB/c mice, and the oral administration of EAE reduced ear epidermal thickness, scratching behavior, and mast cell infiltration. Furthermore, we demonstrated that it activates the intracellular Nrf2/HO-1 signaling pathway, which may have potential therapeutic effects on AD-like lesions.

## Figures and Tables

**Table 1:** Average major organ weight (g) in male and female mice following oral administration of *Erigeron annuus* extract (EAE).

Parameters	Normal	EAE (mg/kg)	
		500	1000
Males			
Heart	0.115 ± 0.021	0.113 ± 0.015	0.120 ± 0.012
Liver	0.978 ± 0.035	1.015 ± 0.061	1.033 ± 0.058
Spleen	0.093 ± 0.008	0.095 ± 0.015	0.090 ± 0.007
Kidney	0.333 ± 0.011	0.335 ± 0.038	0.350 ± 0.017
Lung	0.170 ± 0.036	0.175 ± 0.025	0.170 ± 0.025
Females			
Heart	0.108 ± 0.020	0.100 ± 0.012	0.105 ± 0.009
Liver	0.793 ± 0.029	0.800 ± 0.047	0.803 ± 0.036
Spleen	0.090 ± 0.010	0.088 ± 0.013	0.078 ± 0.004
Kidney	0.245 ± 0.021	0.250 ± 0.025	0.238 ± 0.022
Lung	0.145 ± 0.005	0.140 ± 0.012	0.143 ± 0.015

Values are expressed as mean ± standard deviation (SD). Differences between groups were considered significant when *p* < 0.05. There were no significant (*p* > 0.05) differences between the three groups.

**Table 2:** Hematologic parameters in male and female mice in a subacute toxicity test of EAE.

Parameters	Normal	EAE (mg/kg)	
		500	1000
Males			
WBC (10^3^/µL)	5.113 ± 0.342	5.003 ± 0.328	4.947 ± 0.483
LY (%)	96.343 ± 0.313	96.693 ± 0.054	96.397 ± 1.666
MO (%)	0.833 ± 0.090	0.870 ± 0.179	0.840 ± 0.067
NE (%)	2.167 ± 0.107	2.027 ± 0.131	2.227 ± 0.727
EO (%)	0.167 ± 0.029	0.143 ± 0.021	0.153 ± 0.034
RBC (10^6^/µL)	6.907 ± 0.612	6.873 ± 0.596	7.055 ± 0.154
PLT (10^3^/µL)	836.433 ± 86.753	813.800 ± 80.851	795.675 ± 45.653
HGB (g/dL)	14.777 ± 0.614	14.968 ± 0.956	15.155 ± 0.831
HCT (%)	43.833 ± 2.695	43.600 ± 3.439	45.200 ± 2.239
MCV (fL)	63.600 ± 2.707	63.475 ± 0.750	62.325 ± 2.552
MCH (pg)	21.500 ± 1.042	21.825 ± 0.580	20.900 ± 1.022
MCHC (g/dL)	33.767 ± 0.953	34.350 ± 0.541	33.525 ± 0.432
Females			
WBC (10^3^/µL)	3.650 ± 0.282	3.867 ± 0.212	3.817 ± 0.095
LY (%)	95.850 ± 0.947	96.587 ± 0.764	96.133 ± 0.910
MO (%)	1.300 ± 0.211	1.377 ± 0.437	1.273 ± 0.127
NE (%)	1.697 ± 0.418	1.607 ± 0.450	1.757 ± 0.452
EO (%)	0.227 ± 0.025	0.210 ± 0.051	0.213 ± 0.040
RBC (10^6^/µL)	6.573 ± 0.510	7.080 ± 0.262	6.987 ± 0.262
PLT (10^3^/µL)	857.133 ± 35.171	823.633 ± 65.037	818.100 ± 34.851
HGB (g/dL)	14.120 ± 0.115	15.257 ± 0.794	14.930 ± 0.692
HCT (%)	39.900 ± 0.735	42.767 ± 1.605	42.667 ± 0.785
MCV (fL)	60.500 ± 4.032	61.833 ± 2.517	61.067 ± 1.482
MCH (pg)	21.600 ± 1.715	21.600 ± 1.687	21.433 ± 1.470
MCHC (g/dL)	35.700 ± 0.510	34.833 ± 1.352	35.000 ± 1.558

Values are expressed as mean ± standard deviation (SD). Differences between groups were considered significant when *p* < 0.05. There were no significant (*p* > 0.05) differences between the three groups.

**Table 3:** Serum biochemical parameters in male and female mice in a subacute toxicity test of *Erigeron annuus* extract (EAE).

Parameters	Normal	EAE (mg/kg)	
		500	1000
Males			
ALP (U/L)	43.967 ± 14.281	39.467 ± 3.262	43.767 ± 7.144
ALT (U/L)	42.667 ± 2.357	44.333 ± 2.494	49.000 ± 3.742
AST (U/L)	183.000 ± 3.559	186.333 ± 5.558	191.667 ± 6.182
TB (mg/dL)	1.160 ± 0.008	1.177 ± 0.009	1.177 ± 0.009
TP (g/dL)	5.457 ± 0.054	5.737 ± 0.153	5.717 ± 0.270
ALB (g/dL)	3.060 ± 0.037	3.253 ± 0.116	3.247 ± 0.049
TG (mg/dL)	38.533 ± 0.499	37.167 ± 2.193	40.567 ± 6.975
TC (mg/dL)	112.000 ± 3.742	129.333 ± 10.209	126.267 ± 19.072
LDH (U/L)	416.000 ± 4.546	433.333 ± 23.977	447.333 ± 25.773
Females			
ALP (U/L)	11.133 ± 2.106	11.117 ± 1.899	12.133 ± 1.791
ALT (U/L)	33.000 ± 0.816	31.000 ± 1.633	30.333 ± 0.471
AST (U/L)	118.333 ± 4.497	112.333 ± 10.209	110.867 ± 9.015
TB (mg/dL)	1.133 ± 0.005	1.137 ± 0.005	1.123 ± 0.005
TP (g/dL)	4.997 ± 0.302	4.633 ± 0.139	4.533 ± 0.097
ALB (g/dL)	2.830 ± 0.134	2.787 ± 0.100	2.660 ± 0.090
TG (mg/dL)	54.867 ± 6.715	57.567 ± 8.892	58.033 ± 4.151
TC (mg/dL)	67.467 ± 3.332	62.333 ± 5.526	60.833 ± 2.941
LDH (U/L)	380.667 ± 20.677	372.333 ± 10.873	372.000 ± 6.377

Values are expressed as mean ± standard deviation (SD). Differences between groups were considered significant when *p <* 0.05. There were no significant (*p* > 0.05) differences between the three groups.

## Data Availability

Data is contained within the article.
